# Detection of Vulnerable Coronary Plaques Using Invasive and Non-Invasive Imaging Modalities

**DOI:** 10.3390/jcm11051361

**Published:** 2022-03-01

**Authors:** Anna van Veelen, Niels M. R. van der Sangen, Ronak Delewi, Marcel A. M. Beijk, Jose P. S. Henriques, Bimmer E. P. M. Claessen

**Affiliations:** Heart Center, Department of Cardiology, Amsterdam UMC, University of Amsterdam, Amsterdam Cardiovascular Sciences, 1105 AZ Amsterdam, The Netherlands; a.vanveelen@amsterdamumc.nl (A.v.V.); n.m.r.vandersangen@amsterdamumc.nl (N.M.R.v.d.S.); r.delewi@amsterdamumc.nl (R.D.); m.a.beijk@amsterdamumc.nl (M.A.M.B.); j.p.henriques@amsterdamumc.nl (J.P.S.H.)

**Keywords:** vulnerable plaque, thin-cap fibroatheroma, intracoronary imaging, non-invasive imaging

## Abstract

Acute coronary syndrome (ACS) mostly arises from so-called vulnerable coronary plaques, particularly prone for rupture. Vulnerable plaques comprise a specific type of plaque, called the thin-cap fibroatheroma (TFCA). A TCFA is characterized by a large lipid-rich necrotic core, a thin fibrous cap, inflammation, neovascularization, intraplaque hemorrhage, microcalcifications or spotty calcifications, and positive remodeling. Vulnerable plaques are often not visible during coronary angiography. However, different plaque features can be visualized with the use of intracoronary imaging techniques, such as intravascular ultrasound (IVUS), potentially with the addition of near-infrared spectroscopy (NIRS), or optical coherence tomography (OCT). Non-invasive imaging techniques, such as computed tomography coronary angiography (CTCA), cardiovascular magnetic resonance (CMR) imaging, and nuclear imaging, can be used as an alternative for these invasive imaging techniques. These invasive and non-invasive imaging modalities can be implemented for screening to guide primary or secondary prevention therapies, leading to a more patient-tailored diagnostic and treatment strategy. Systemic pharmaceutical treatment with lipid-lowering or anti-inflammatory medication leads to plaque stabilization and reduction of cardiovascular events. Additionally, ongoing studies are investigating whether modification of vulnerable plaque features with local invasive treatment options leads to plaque stabilization and subsequent cardiovascular risk reduction.

## 1. Introduction

Coronary artery disease (CAD) is one of the most common diseases in developed countries and has a high morbidity and mortality [1]. Although mortality rates among patients with ischemic heart disease have declined over the last decades, the occurrence of acute coronary syndrome (ACS) is still unchanged and remains responsible for billions of health care expenditures worldwide [2]. Therefore, early identification of patients vulnerable to ACS could lead to a reduction of morbidity and economic burden.

The majority of ACS arises from rupture or erosion of a coronary plaque leading to (sub)acute thrombosis [3,4]. Together with vasoconstriction and increased coagulability, thrombosis may lead to acute cessation of the coronary blood flow and subsequent myocardial ischemia [5]. Some coronary plaques are particularly prone to rupture as first described in 1988 by Muller et al. [5]. “Vulnerable plaques” are often non-obstructive and cause limited lumen compromise [4,6]. However, the vulnerable plaques are characterized by specific high-risk features that increase the risk for plaque rupture and are often referred to as lipid-rich plaque or thin-cap fibroatheroma (TCFA) [7,8].

Different invasive and non-invasive imaging modalities are able to detect the vulnerable plaques. The imaging modalities each specifically visualize different vulnerable plaque features. The gold standard for vulnerable plaque detection is invasive coronary imaging, such as intravascular ultrasound (IVUS) and optical coherence tomography (OCT), since these techniques are most established and provide detailed information on plaque morphology and composition. But non-invasive imaging techniques such as computed tomography coronary angiography (CTCA), cardiac magnetic resonance (CMR) imaging, and nuclear imaging techniques can also be used to detect vulnerable plaques. In this review, we discuss the currently available invasive and non-invasive imaging techniques to detect vulnerable plaques. In particular, we will focus on the detection of rupture-prone plaques.

## 2. Histopathological Features of Vulnerable Plaques

**Figure 1:**
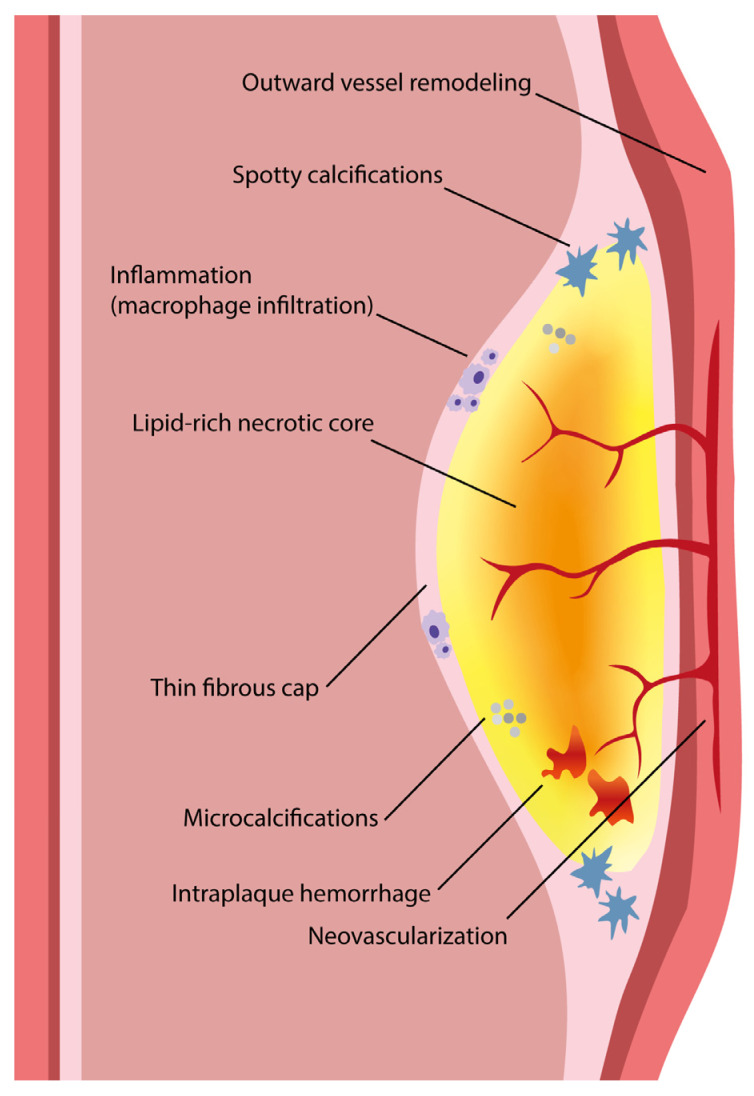
The vulnerable plaque consists of a large lipid-rich necrotic core with a thin fibrous cap (<65 μm). Several plaque features can be present that are associated with increased risk for cardiovascular events, including outward vessel remodeling, microcalcifications and spotty calcifications, hemorrhage, neovascularization, and inflammation. Image adapted from Van Veelen et al. Reviews in Cardiovascular Medicine 2022. CC-BY [4.0] [9].

The histopathological characteristics of vulnerable plaques have been studied in deceased humans and animals. Autopsy studies in patients who died from suspected sudden coronary death reported that ruptured plaques consisted of a large lipid-rich core with central necrosis [8]. The formation of these lipid-rich plaques comprises a series of processes involving influx and expression of different cell types in the arterial wall. These individual plaque processes may serve as targets for the available imaging modalities. Figure 1 provides an overview of the vulnerable plaque features.

The first stage of atherosclerosis arises when minimal spontaneous injury to the coronary endothelium occurs, usually at specific vulnerable areas in the coronary artery where the unidirectional laminar flow is disturbed, for instance near side branches, bifurcations, or at bending points [10,11,12,13]. Contrarily, where low shear stress predisposes vulnerable plaque formation, high shear stress could ultimately precipitate plaque rupture [14,15]. Through the injured endothelium, circulating plasma low-density lipoprotein (LDL) particles enter the intima where they accumulate and become oxidized [16,17]. The LDL particles recruit monocytes into the vessel wall, where they differentiate into macrophages. The oxidized LDL binds to the macrophages which then transform into so-called foam cells (i.e., lipid-laden macrophages) [18]. The foam cells secrete proinflammatory enzymes and cytokines, leading to activation of the immune system and attraction of immune cells that further accelerate the inflammatory process [19,20]. As a consequence of inflammation, smooth muscle cells proliferate, and additional smooth muscle cells are recruited from the media [21,22]. Moreover, a calcification cascade is initiated by the inflammatory cytokines which finally leads to the formation of microcalcifications and spotty calcifications [23].

As the atherosclerotic process evolves, the foam cells continue to take up the excessive LDL, leading to foam cell necrosis or apoptosis and an extracellularly accumulation of lipid, which forms the necrotic lipid-rich core [24]. Continued influx of plasma LDL cholesterol will lead to cholesterol crystal formation [18]. Rupture-prone plaques also often manifest neovascularization and subsequent intraplaque hemorrhage, which could further contribute to plaque vulnerability and enlargement of the lipid-rich core, since erythrocytes are rich in cholesterol [25,26]. Intraplaque hemorrhage is considered to be an important factor of plaque instability. The smooth muscle cells in the intima produce an extracellular matrix consisting of proteoglycans, collagen, and elastine, that forms a fibrous cap to cover the core of necrotic foam cells [22]. This stage is often irreversible and accompanied by outward vascular remodeling, preventing the cell accumulation in the intima to cause lumen compromise [27]. In the subsequent stages of vulnerable plaque formation, the necrotic core further enlarges. This promotes thinning of the fibrous cap, by the loss of smooth muscle cells and continued influx of macrophages that degrade the cap matrix [28,29]. Cap thickness is generally less than 65 μm in ruptured plaques [30,31].

All vulnerable plaque features were summarized in a consensus document by Naghavi et al., in 2003 [32,33]. The aforementioned high-risk plaque features, i.e., large lipid core with thin fibrous cap, the presence of active inflammation (macrophage or T-cell infiltration), together with denudation of the endothelium were defined as major criteria. Minor features are (1) the presence of calcified nodules that protrude through the cap, which may cause cap rupture, (2) a (glistening) yellow aspect on angioscopy, which could imply a high burden of lipid with a thin cap; (3) intraplaque hemorrhage, indicating plaque instability; and (4) endothelial dysfunction. The definition by Naghavi et al. also considers lesions with >90% diameter stenosis as vulnerable plaques, since rapid progression would also lead to clinical events.

## 3. Intracoronary Imaging Modalities

Historically, coronary angiography (CAG) is the gold standard for coronary plaque assessment. It is used to evaluate luminal stenosis grade and to identify coronary calcifications; however, it is unable to identify the high-risk plaque features that predict plaque rupture [4]. As discussed above, lesions that eventually may cause a coronary occlusion in the setting of ACS are often unsuspicious on previous CAG, typically showing low stenosis grades [4]. Therefore, intracoronary imaging modalities that allow in vivo assessment of plaque morphology, tissue composition, or the presence of inflammation, are required to identify the vulnerable plaques. All features of the intracoronary imaging modalities are summarized in Table 1.

### 3.1. Intravascular Ultrasound

IVUS is a catheter-based imaging technique using ultrasonography that allows visualization of all layers of the coronary artery wall and characterization of plaque morphology [34,35]. The transducer is located at the tip of the IVUS catheter which is retracted through manual or automated pullback, with speeds varying between 0.5–1.0 mm/s to 10 mm/s. Intracoronary images were traditionally derived from ultrasound frequencies in the range of 20–40 MHz, which resulted in a limited axial resolution of around 150 μm [36]. However, novel high-definition (HD) IVUS systems use frequencies up to 60 MHz, gaining a much lower axial resolution that even approaches that of OCT, such as 40 μm with the HDi™ HD IVUS System (ACIST Medical Systems, Inc., Eden Prairie, MN, USA) or even 20–22 μm with the OptiCross™ HD system (Boston Scientific, Marlborough, MA, USA) or the Makoto IVUS Imaging System (Nipro, Bridgewater, NJ, USA). These IVUS systems with improved resolution could potentially improve the diagnostic value of IVUS to identify vulnerable plaques [37]. An advantage of IVUS is that it does not require blood clearance from the vessel lumen or additional contrast use, and it has the ability to penetrate through the vessel wall. The penetration depth of IVUS is around 4–8 mm, enabling visualization of all layers of the coronary artery (including the external elastic membrane and surroundings) and the presence of positive vessel remodeling, which is associated with plaque instability [27,38,39].

**Figure 2:**
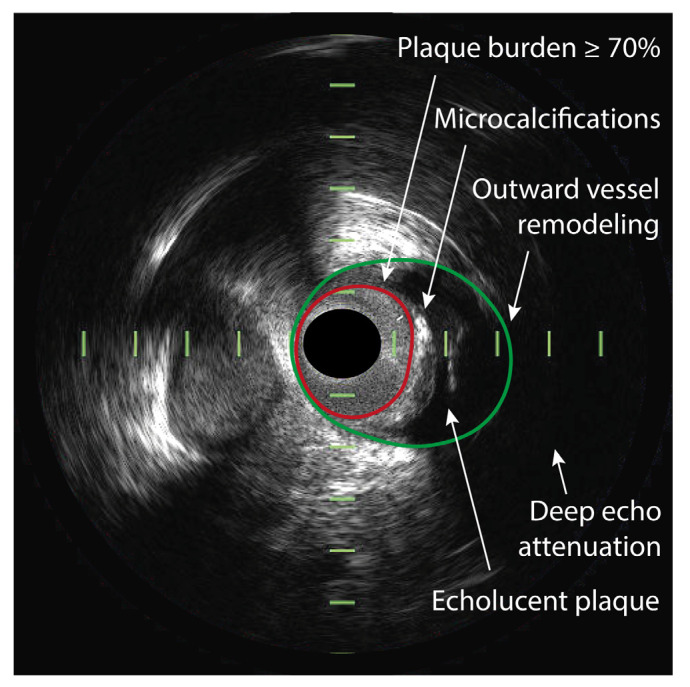
Vulnerable plaque on IVUS. An intravascular ultrasound (IVUS) cross-section of the coronary artery demonstrating the vulnerable plaque features that can be visualized with IVUS. The plaque demonstrates a plaque burden that is greater than 70%, measured as the external elastic membrane (EEM) area (green line) minus the luminal area (red line), divided by the EEM. The plaque appears echolucent, indicating the presence of a large lipid core and deep echo attenuation is visible. Furthermore, microcalcifications and outward vessel remodeling can be observed.

Grayscale IVUS is the conventional ultrasonography method that results in black and white IVUS images; see Figure 2. It permits assessment of lumen and vessel dimensions and plaque morphology. Additionally, the plaque burden can be measured, as (external elastic membrane area—lumen area)/external elastic membrane area × 100%, which is an approximation of the atheroma volume.

Information on plaque composition can be derived from the echodensity of the plaque compared to the echodensity of the adventitia, where echogenic (lighter) areas with acoustic shadowing indicate the presence of calcifications and echolucent (darker) areas indicate soft plaque, e.g., consisting of fibrous or lipid tissue [40,41]. Moreover, echolucent plaques with larger lipid-rich necrotic cores are often accompanied by deep ultrasound attenuation [42,43]. Moreover, as mentioned above, another important feature of a vulnerable plaque is the presence of intraplaque hemorrhage. HD IVUS is a useful tool to identify intraplaque hemorrhage, which appears as an echolucent area with well-delineated borders, that has a crescent shape circumscribed within the plaque [44,45,46].

Post-processing IVUS modalities have been developed to enhance plaque composition information using the radiofrequency backscattered ultrasound signals (IVUS-RF); examples are virtual histology (VH-IVUS) and integrated backscatter (IB-IVUS). The IVUS backscatter contains specific information about tissue composition which can be converted into a colored tissue map of the coronary artery wall using mathematical manipulation [47]. This yields an in-vivo predictive accuracy of 88% to detect a necrotic region, as well as 87% and 97% for fibrous/fibro-fatty and calcified regions, respectively [48].

Guidelines currently recommend the use of IVUS for the grading of stenosis severity and guidance of stent implantation [49,50,51], but it also allows for the evaluation of non-obstructive vulnerable plaques. Nicholls et al. combined serial IVUS images of 4137 patients that participated in six clinical trials to evaluate the prognostic value of IVUS-derived parameters to predict outcome [52]. They found that the plaque burden, as measured with IVUS at baseline, was incrementally predictive for the occurrence of myocardial infarction, coronary revascularization, or a composite endpoint of major adverse cardiovascular events (MACE). Since the exact cap thickness cannot be measured with IVUS due to the limited axial resolution, an alternative definition for TCFA has been proposed for VH-IVUS by Rodriguez-Granillo et al. [53]. This definition comprised plaques with a plaque burden of >40% and a large necrotic-rich core (i.e., >10% of the cross-sectional area), without apparent overlying fibrotic tissue [53,54]. These IVUS-derived TCFAs are more often seen in patients with ACS than patients with stable CAD and more often found in the proximal segments of the coronary arteries [53]. The sensitivity and specificity to identify TCFA with VH-IVUS based on this definition has been reported to be 64% and 78% compared with histology [55].

The PROSPECT study was the first natural history study that prospectively evaluated which IVUS-RF plaque features predict clinical outcome [56]. A total of 697 patients with ACS underwent both grayscale and IVUS-RF after successful PCI of the culprit lesion, and patients were clinically followed for at least 2 years. MACE, defined as cardiac death, cardiac arrest, myocardial infarction, or rehospitalization for unstable or progressive angina, was registered and events were divided as being related to the index lesion (culprit) or to a non-culprit lesion. Half of the observed MACE was culprit-related, and half was caused by a non-culprit lesion (in total, 11.6% non-culprit events during 3.4 years of follow-up). These non-culprit events mostly arose from non-obstructive lesions with a mean diameter stenosis of 32%. The study confirmed that IVUS was able to predict the risk for non-culprit MACE. High-risk IVUS features for long-term non-culprit events were plaques that were scored as TCFA based on the aforementioned VH-IVUS definition (threefold increased risk) and plaques with a plaque burden greater than 70% (fivefold increased risk). Additionally, the more recent PROSPECT II study was performed in a total of 898 patients with recent myocardial infarction [57]. All patients underwent three-vessel IVUS with near-infrared spectroscopy (NIRS). A large plaque burden was again a strong predictor of future MACE within 4 years of follow-up.

### 3.2. Optical Coherence Tomography

**Figure 3:**
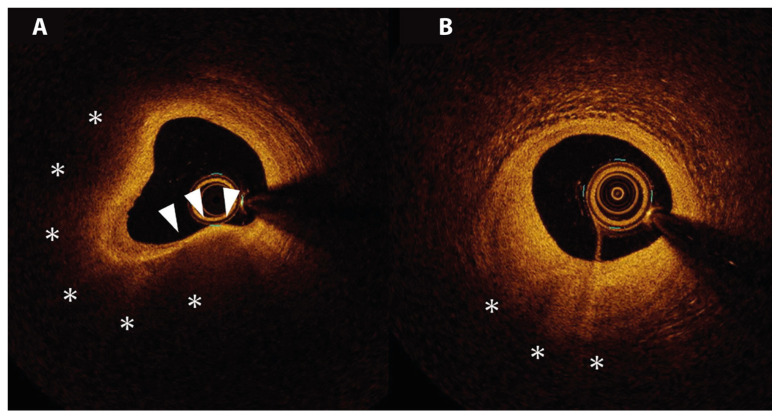
Vulnerable plaque on OCT. A cross-section of the coronary artery with OCT demonstrates a low-signal region, marked with asterisks, corresponding with a lipid-rich plaque. The overlying bright structure corresponds with the fibrous cap (arrowheads). (**A**) displays a lipid-rich plaque with a thin fibrous cap (i.e., thin-cap fibroatheroma). (**B**) displays a lipid-rich plaque with thick fibrous tissue overlaying the lipid-rich core. Image obtained from Muramatsu Y. et al., IJC Heart & Vasculature 2019. CC-BY [4.0] [71].

OCT can be considered an optical analogue of IVUS, which uses the optical echoes of near-infrared light instead of ultrasonography. The rotating OCT probe located at the tip of the OCT catheter contains an optical fiber which emits near-infrared light, and a detector. During an automated pullback with speeds up to 20 mm/s, near-infrared light is emitted from the probe and then reflected of the arterial wall. The backscatter is gathered by the detector and translated into an image based on the duration of reflection and intensity of the backscattered light [58]. Since red blood cells cause scattering of the near-infrared light, blood clearance of the vessel by contrast injection is necessary for accurate images. The contrast injection can be performed manually or automatically. Due to the use of light, OCT produces high-resolution images with axial resolutions as high as 10–15 μm [59]. The excellent axial resolution qualifies OCT as the sole available imaging modality to measure the cap thickness; see Figure 3 [60].

The signal intensity that appears on the OCT image correlates with tissue composition. Lipid-rich tissues appear as low-signal areas with diffuse borders and fibrous tissues as signal-rich areas [61]. However, calcified plaques also appear as low-signal areas, although with sharp borders, but this could hinder adequate distinction between these two plaque types. In a comparison study between IVUS and OCT, the high-risk plaque features that were detected with IVUS, such as calcifications and echolucent regions indicating lipid pools, were detected with OCT as well, and OCT was even able to visualize more lipid pools than IVUS [59]. A major disadvantage of OCT is the limited tissue penetration, i.e., only 1–2 mm, which hampers visualization of the external elastic membrane in regions with large atherosclerotic plaques. As a consequence, it has been challenging to evaluate the plaque burden with OCT: the IVUS feature that was the most accurate predictor for adverse cardiac events in the PROSPECT studies [56,57]. Alternatively, studies used the plaque free wall angle or the lipid arc as a surrogate for plaque burden [62,63]. More recently, an algorithm has been presented that enhances the external elastic membrane, enabling more accurate plaque burden measurement similar to that with IVUS [64]. Other vulnerable plaque features that can be visualized with OCT include neovascularization, intraplaque hemorrhage, calcifications, and cholesterol crystals [65]. Inflammation of the vulnerable plaque can be visualized and quantified by measuring the macrophage infiltration in the fibrous cap [66,67]. High sensitivities and specificities have been reported of OCT to detect lipid-rich plaques, validated with autopsy specimens (i.e., 90–94% and 90–92%, respectively) [68].

The COMBINE OCT-FFR trial by Kedhi et al. investigated the prognostic impact of the presence of a TCFA as identified with OCT in patients with diabetes mellitus [69]. From the total cohort of 550 patients, 25% had a hemodynamically insignificant TCFA (i.e., fractional flow reserve >0.80), and it was observed that these patients had a fivefold higher risk for adverse cardiac events within 18 months, consisting of cardiac mortality, target vessel myocardial infarction, target lesion revascularization, or unstable angina requiring hospitalization, compared with patients without TCFA. In the CLIMA study, a total of 1003 patients underwent OCT imaging of the untreated proximal left anterior descending artery and were clinically followed-up for 1 year [70]. The presence of a minimal luminal area <3.5 mm^2^, fibrous cap thickness <75 μm, lipid arc extension >180°, and OCT-derived macrophages were all individually predictive for the occurrence of the composite endpoint of cardiac death and myocardial infarction in the target segment. Combining these OCT parameters yielded an hazard ratio of 7.5. Thus, OCT seems to be able to identify vulnerable plaques that are at increased risk to cause future cardiac events, and future prospective studies are warranted to demonstrate the clinical impact of OCT-derived TCFAs in an all-comers patient population.

### 3.3. Near-Infrared Spectroscopy

NIRS is a technique that is often used in physical sciences and other medical disciplines to determine the chemical composition of certain substances. To aid the detection of TCFAs, efforts were made to develop a safe and accurate catheter-based system for intracoronary use [72]. The technique is able to differentiate between various substances based on the unique patterns in which they absorb and scatter the near-infrared light. A near-infrared fiber-optic probe was constructed to detect intracoronary infrared wavelengths and an algorithm was developed that successfully detected the wavelengths corresponding with cholesterol. In 1993 Cassis and Lodder demonstrated that this algorithm was able to visualize lipid deposits in rabbit aortas [73]. In 2002, Moreno et al. first reported the application of NIRS for the detection of lipid-rich plaques in human aorta specimen [74]. Validated with histology, the authors found a sensitivity and specificity of 90% and 93% for lipid pool and 77% and 93% for thin caps, respectively.

**Figure 4:**
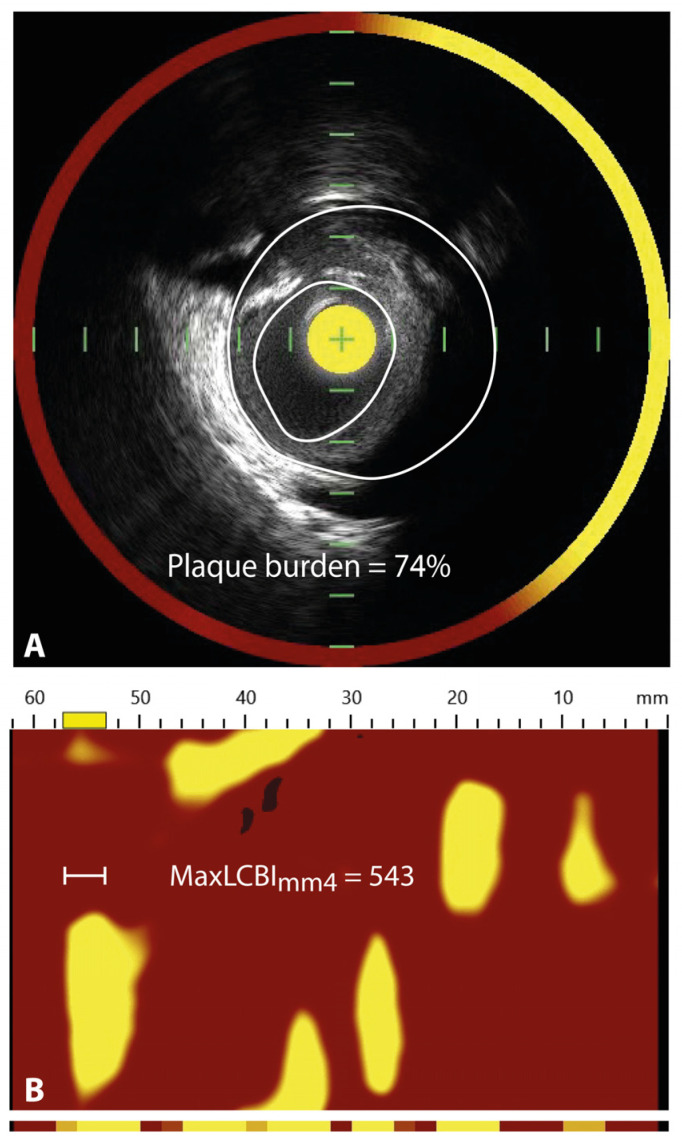
Vulnerable plaque on NIRS-IVUS. (**A**) displays an intravascular ultrasound (IVUS) image, which demonstrates an echolucent plaque with deep echo attenuation and a large plaque burden of 74%. The red-to-yellow colored ring corresponds with near-infrared spectroscopy (NIRS) data. The ring colors yellow at the site of the soft plaque, indicating that the plaque corresponds with a high probability for lipid core. In (**B**), the corresponding NIRS chemogram is displayed, where a maximum lipid-core burden index in a segment of 4 mm (maxLCBImm4) is detected of 543 at around 55 mm of the pullback, corresponding with the definition of a lipid-rich plaque (maxLCBImm4 > 400), based on the LRP study [79]. Image adapted from Van Veelen et al. Reviews in Cardiovascular Medicine 2022. CC-BY [4.0] [9].

To provide both compositional and structural data, an integrated NIRS-IVUS catheter has been developed enabling co-registration of IVUS and NIRS images (Infraredx Inc., a Nipro Company, Bedford, MA, USA); Figure 4. Alternatively, there have been reports about co-registration of NIRS with OCT as well [75,76]. The addition of NIRS to IVUS improves the ability to detect lipid-rich plaques and it gains extra information since NIRS is able to accurately penetrate through calcium and stent struts [77,78]

The NIRS-IVUS catheter is similar in size as the IVUS catheters, and compatible with five French guiding catheters. It comprises both a NIRS laser as well as an IVUS probe and, through an automated or manual pullback and rotational tip, approximately 1300 NIRS spectra per millimeter are acquired. Spectrums receive a probability score varying between 0 to 1 for the likelihood to contain lipid, validated with a large histology autopsy study in humans [80]. This probability is translated into a colored pixel, generating a two-dimensional colored map of the coronary artery, the so-called chemogram. The pixels are colored red for a probability of 0 for lipid core, which shifts to orange when the probability is >0.60 and to yellow for high probability (>0.98). Apart from the visual information from the chemogram, a quantitative measure of the lipid burden is also offered, called the lipid-core burden index (LCBI). This corresponds with the amount of yellow on the chemogram and is computed as the fraction of valid yellow pixels within a region of interest, multiplied by 1000 [81].

In 2013, Madder et al. performed NIRS within the culprit vessel of patients with ST-segment elevation myocardial infarction and the maximum LCBI per 4 mm (maxLCBI_4mm_) within culprit segments was compared to non-culprit segments and autopsy segments that were histologically free from lipid cores [82]. The culprit segments had much higher maxLCBI_4mm_ than the control segments, i.e., sixfold higher than non-culprit segments and 87-fold higher than lipid core free segments. A cut-off value of maxLCBI_4mm_ > 400 was found to accurately distinguish the culprit segments from the lipid core free autopsy segments, which yielded a sensitivity of 85% and a specificity of 98%. Similar outcomes were observed in patients with non-ST-segment elevation myocardial infarction or unstable angina [83]. Apart from the capability of identifying culprit lesions in patients with ACS, Oemrawsingh et al. were the first to demonstrate that NIRS was also able to predict future culprit lesions [84]. In their single center study, NIRS was performed in non-culprit arteries in a population consisting of both patients with stable CAD and patients with ACS. Patients that had a LCBI above the median had a fourfold risk to experience the composite endpoint of all-cause mortality, nonfatal ACS, stroke, and unplanned coronary revascularization during 1 year follow-up. These results were later confirmed by imaging studies from Madder et al., and Schuurman et al., who demonstrated the prognostic impact of lipid-rich plaques for clinical outcome [85,86].

The largest study to date that demonstrated the clinical value of NIRS-IVUS was the LRP study where a total of 1563 patients with suspected CAD between 2014 and 2016 underwent three-vessel NIRS-IVUS after successful stenting [79]. Patients were clinically followed for 2 years and a total of 9% non-culprit MACE was observed. MACE consisted of cardiac death, cardiac arrest, non-fatal myocardial infarction, acute coronary syndrome, revascularization by coronary artery bypass grafting or PCI, and readmission to hospital for angina with more than 20% diameter stenosis progression related and unrelated to the treatment at index procedure. An increase of 100 units of the maxLCBI_4mm_ increased the risk with 18% for patients to experience non-culprit MACE. Moreover, the aforementioned threshold of maxLCBI_4mm_ > 400 identified non-culprit plaques that were at fourfold risk of causing MACE.

The PROSPECT II study (vide supra) suggested that a lower cut-off value for maxLCBI_mm4_ could increase the sensitivity of NIRS [57]. The study defined lipid-rich plaques as plaques with maxLCBI_4mm_ within the highest quartile, which was 325. This resulted in an unadjusted sevenfold increased risk for lipid-rich plaques to cause MACE.

### 3.4. Angioscopy

An intracoronary imaging technique that is probably closest to in vivo imaging of the coronary artery wall is angioscopy, where a direct visualization of the luminal surface is provided through optical fibers and projected white light [87]. The coronary angioscope is advanced via a delivery catheter, which carries a soft atraumatic balloon proximal from the angioscope. To obtain a clear image of the vessel wall, the balloon is inflated to occlude blood flow, followed by a saline injection. Angioscopy only visualizes the intimal layer of the vessel wall, allowing detection of irregularities within the endothelium, such as ulceration or fissures, as well as thrombus. Vulnerable plaque assessment with angioscopy is based on plaque color. Normal coronary artery surface appears white, while yellow is associated with the presence of atheroma [88,89]. The intensity of yellow provides information on cap thickness, since the more yellow the surface appears, the thinner the fibrous cap is believed to be. The yellow intensity is scored by the operator, which requires expert knowledge, although automated scoring algorithms are under development [90]. In a comparison study between OCT and angioscopy, it was found that there was an inverse relationship between the intensity of yellow and the fibrous cap thickness [91]. The authors considered caps with a thickness of 110 μm as thin caps, which could be detected with angioscopy based on yellow intensity with a sensitivity and specificity of respectively 98% and 96% in this small cohort [91]. The intensity of yellow was scored from grade 0 for white to grade 3 for dark yellow, and the results demonstrated that plaques that appear with medium (grade 2) or dark yellow (grade 3) have a cap thickness of 72 and 40 μm, respectively. Ueda et al. angioscopically investigated 10 patients with acute myocardial infarction and found that the culprit plaques appeared as yellow plaques with thrombus, supporting the belief that these plaques represent ruptured vulnerable plaques [92].

Since coronary angioscopy is limited by the need for balloon occlusion, which induces ischemia, and the inability to assess the inside of the arterial wall, this imaging technique is not often used in the catheterization laboratory, and it has no specific place in the guidelines. However, novel angioscopy systems are under development that would enable the visualization of the vessel wall using a flushing system without the need for proximal balloon occlusion [93,94].

### 3.5. Thermography

Thermography is an over-the-wire catheter-based system that contains a so-called thermistor with a thermal resolution of 0.0001 °C and a spatial resolution of 0.5 mm [95]. Thermography can be used to visualize inflammation in the vulnerable plaque. The inflammatory process produces heat, which results in local temperature elevations at the plaque surface [29,96]. This local temperature heterogeneity can be detected with a thermography catheter [96]. In an animal study, temperature heterogeneity was present in the aortas of cholesterol-fed rabbits, but not in controls, and it was an indicator for the presence of macrophages [97]. In humans, this thermal heterogeneity was indeed present in two-thirds of patients presenting with acute myocardial infarction and not in control patients with normal coronary arteries [98]. The temperature difference of the culprit plaque, compared with the background temperature, was greatest in patients presenting with acute myocardial infarction compared with patients presenting with (un)stable angina [98]. Subsequently, the same research group investigated whether the technique would also be able to predict future cardiac events. The investigators performed thermography after successful PCI in 86 patients presenting with ACS and stable angina and found that the presence of local temperature elevations was associated with adverse clinical outcome after 18 months [99]. Around 40% of patients with a temperature difference of >0.5 degrees Celsius between the atherosclerotic plaque and the healthy vessel wall experienced adverse events, while this was only 7% in patients with a temperature difference of <0.5 °C. Additionally, thermography was used to visualize the anti-inflammatory effects of statin treatment by demonstrating a temperature decrease in patients that received atorvastatin and could therefore also be of use to evaluate disease progression or regression [100].

Although the technique seemed promising for visualizing one of the hallmarks of vulnerable plaques, it is limited by the need for vessel wall contact by the thermography catheter, hereby possibly inducing vessel injury. Moreover, Cuisset et al. reported in 2009 that temperature changes are closely related to changes in coronary pressure and flow [101]. The authors performed a temporary balloon occlusion creating a low pressure–low flow situation which resulted in a temperature decrease distally from the occlusion, indicating that the abovementioned results could be inaccurate due to pressure and flow artifacts.

## 4. Non-Invasive Imaging Modalities

Although various intracoronary imaging modalities exist that can accurately visualize several high-risk plaque features, detection of the vulnerable plaque with non-invasive imaging modalities would be ideal for screening without the need for invasive catheterization. Additionally, non-invasive vulnerable plaque imaging could also serve as a modality to evaluate plaque regression in trials that evaluate treatment strategies for the vulnerable plaque. Non-invasive cardiovascular imaging modalities are established in demonstrating the presence (e.g., coronary calcium) or the consequence of CAD (e.g., ischemia or infarction on perfusion imaging), but efforts are now made to detect the asymptomatic non-obstructive vulnerable plaques as well. Table 2 summarizes the features of the available non-invasive imaging modalities.

### 4.1. Computed Tomography Coronary Angiography

CTCA is a non-invasive imaging modality with relatively low radiation exposure that provides a high spatial resolution. CTCA is highly accurate in the detection of coronary calcium and obstructive CAD with reported sensitivity of 98–99% and specificity of 64–91% [102,103]. This high accuracy also allows for the measurement of several plaque parameters, including luminal area, stenosis grade, and plaque volume. These parameters have been compared with IVUS in a large meta-analysis comprising 42 studies which demonstrated that the CTCA measurements corresponded well with those on IVUS [104]. Automated measurement of CTCA-derived plaque parameters is currently under development, and it demonstrated comparable outcomes to the semi-automated measurements [105].

**Figure 5:**
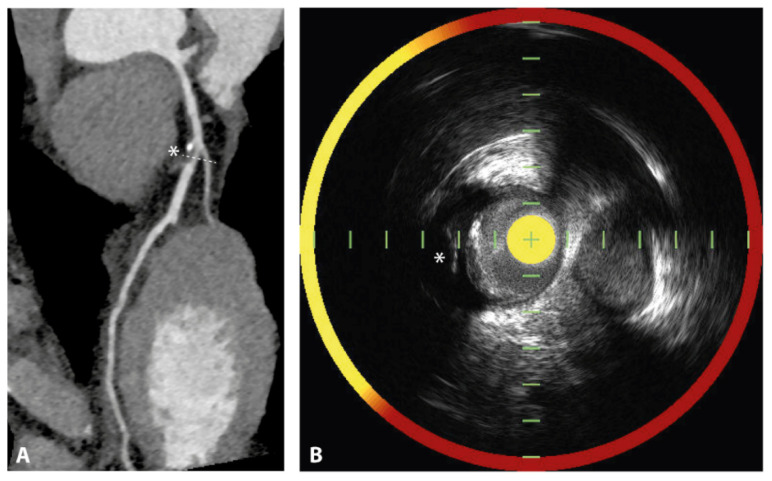
Vulnerable plaque on CTCA. (**A**) displays the left anterior descending artery on computed tomography coronary angiography (CTCA) of a patient with stable angina pectoris. A low-attenuation plaque is demonstrated with spotty calcification. (**B**) displays the intravascular ultrasound (IVUS) images with near-infrared spectroscopy (NIRS) of the same patient, corresponding with the cutline in (**A**) around the bifurcation. The IVUS image displays an echolucent plaque (*) with deep echo attenuation and small calcium deposits. The NIRS chemogram colors yellow, indicating the presence of a large lipid core. Image obtained from Van Veelen et al. Reviews in Cardiovascular Medicine 2022. CC-BY [4.0] [9].

Plaque differentiation can be performed on CTCA by evaluation of the CT attenuation, expressed in Hounsfield Units (HU). This correlates with echogeneity on IVUS [106]. Plaques with high CTCA attenuation (HU > 500) correspond with calcified plaques, while low-attenuation plaques (HU < 30) correspond with lipid-rich plaques on (VH-)IVUS, see Figure 5 [107]. Leschka et al., performed an ex vivo validation study, where contrast-enhanced CTCA in 25 human heart specimens was compared with histopathology [108]. High sensitivities and specificities were found for the identification of advanced calcified or mixed plaques, but CTCA was also able to distinguish the early non-calcified plaques with excellent sensitivity (100%) and acceptable specificity (72%).

In a study by Motoyama et al. evaluating 71 patients undergoing CTCA, plaque features of patients with stable angina pectoris and patients with ACS were compared [109]. The features that were most associated with ACS were plaques with (1) low CT-attenuation, (2) positive vessel remodeling, and (3) spotty calcifications (i.e., punctate calcifications < 3 mm [110]). A combination of those three parameters had a strong positive predictive value of 95% and when these three predictors were absent, no plaques associated with ACS were missed (i.e., negative predictive value of 100%). Hereafter, Motoyama et al. prospectively tested the predictive value of these CTCA-derived plaque features in a study of 1057 patients who underwent CTCA and were followed-up for 27 months [111]. The remodeling index on CTCA was calculated as the lesion diameter divided by the reference diameter proximal from the lesion, which can be used to evaluate the severity of remodeling. The definition of positive remodeling is a remodeling index of >1.1 (i.e., >110%) [111]. Coronary plaques that demonstrated low CT attenuation and positive vessel remodeling had a 23-fold higher risk to cause ACS than plaques that did not demonstrate these features. Spotty calcifications, which were an important predictor for ACS in the previous study, did not have a statistically significant predictive value. Nonetheless, they were numerically more present in low-attenuation plaques with positive vessel remodeling resulting in ACS than in plaques with the same two features that did not result in ACS.

**Figure 6:**
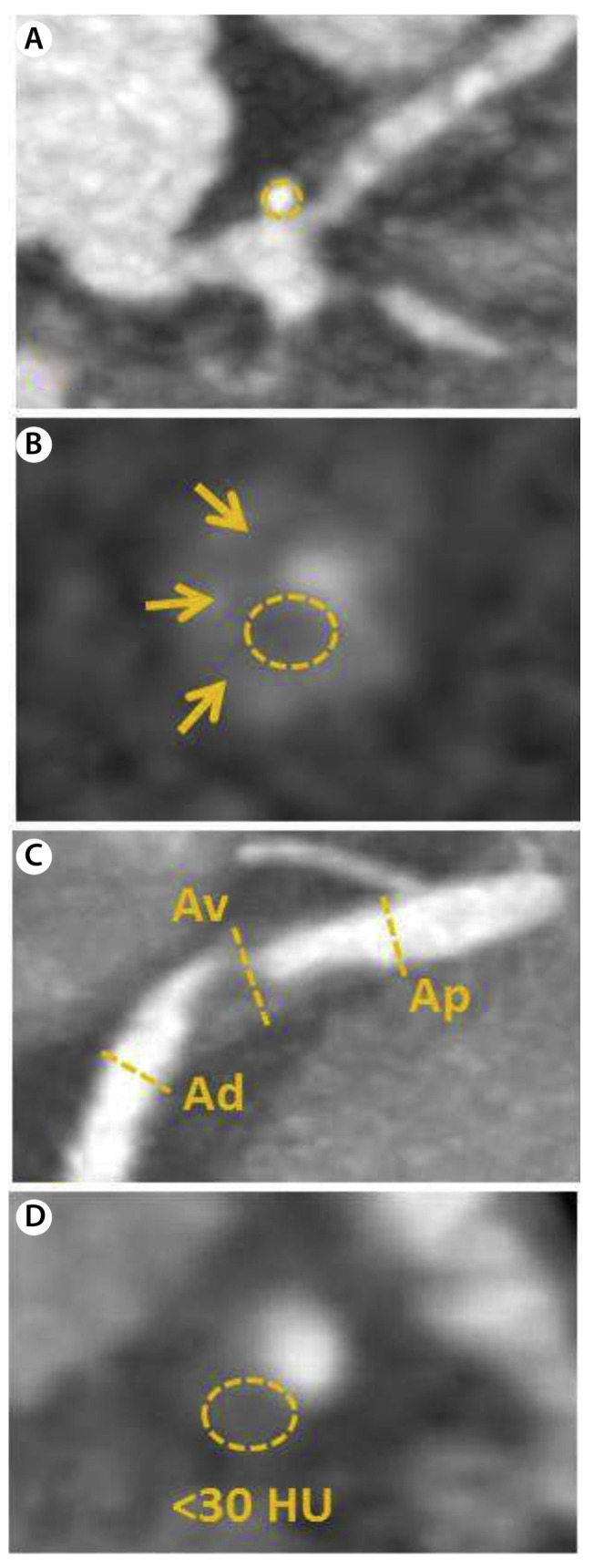
High-risk plaque features on CTCA according to CAD-RADS™: Coronary Artery Disease-Reporting and Data System. (**A**) demonstrates spotty calcifications; (**B**) demonstrates the napkin-ring sign, i.e., plaque with low attenuation in the center and a peripheral rim of high attenuation (indicated with arrows); (**C**) demonstrates positive remodeling, which is present if the ratio of the vessel diameter at the location of the plaque (Av), in relation to the vessel diameter proximally (Ap) and distally from the plaque (Ad) is greater than 1.1; (**D**) demonstrates a low-attenuation plaque, with Hounsfield Units (HU) of <30. Reprinted from Cury et al. [116], with permission from Elsevier.

In addition to the level of CT attenuation in the plaque, the pattern of attenuation has also been shown to be predictive for the presence of vulnerable plaque. The napkin-ring sign appears on the coronary artery cross-section as a low-attenuation plaque core in contact with the lumen, surrounded by a bright, ring-shaped, high-attenuation rim area. The presence of the napkin-ring sign is associated with the presence of TCFA on OCT [112,113,114]. Otsuka et al. demonstrated in a prospective study of 895 patients undergoing CTCA with clinical follow-up for over 2 years, that the napkin-ring sign was an additional strong predictor for an ACS event, consisting of cardiac death, nonfatal myocardial infarction, or unstable angina—independent from the previously demonstrated CTCA features of low-attenuation and positive remodeling [115]. The presence of a napkin-ring sign was associated with a sixfold increased risk for a cardiac event. Figure 6 summarizes all high-risk plaque features that can be visualized with CTCA.

A novel CT feature is the measurement of fractional flow reserve. This provides additional information on the hemodynamic effects of coronary plaques and is able to reduce the need for invasive coronary angiography after CTCA [117]. The role of fractional flow reserve for the detection of vulnerable plaques seems questionable, since most ACS arises from non-significant coronary plaques. However, an impaired CT-derived fractional flow reserve was associated with positive remodeling and the presence of spotty calcifications—high-risk features that are indicative for a vulnerable plaque on CTCA [118]. This suggests a potential additional diagnostic value of CT-derived fractional flow reserve.

Plaque regression or progression can also be visualized with CTCA. In statin trials, the reduction of non-calcified plaque burden in patients on a statin regime can be successfully calculated with CTCA [119,120,121]. Moreover, in a sub-study of the PROSPECT trial, a total of 32 patients underwent CTCA at baseline and 3 years after their index procedure and an increase of plaque volume was observed, as well as the occurrence of compensatory positive remodeling [122]. These results demonstrate that CTCA may be utilized for studies on lipid-rich plaque interventions.

### 4.2. Cardiovascular Magnetic Resonance Imaging

Cardiovascular magnetic resonance (CMR) imaging has a comparable spatial resolution to CTCA but does not require the use of ionizing radiation. Moreover, the soft tissue characterization with CMR is superior to that of CTCA. In carotid atherosclerosis, CMR has been able to differentiate between varying plaque types [123]. Using a series of scans with multiple contrast-weighted images, different plaque components can be visualized, such as the lipid content, intraplaque hemorrhage, neovascularization, and fibrous cap thickness [123,124,125,126,127]. The application of CMR for the detection of coronary vulnerable plaques has not yet been established and could be limited by cardiac and pulmonary movement.

Karolyi et al. performed CMR on 28 coronary artery specimens with proven CAD and used varying sequences to visualize the coronary plaques [128]. Three plaque types were distinguished: lipid-rich plaques, calcified plaques, and fibrotic plaques. Calcified plaques appear hypointense on T1-weighted images but not on the ultrashort echo time images, and lipid-rich plaques appear as hypointense areas on T2-weighted images. Plaques that were isointense on all image series indicated fibrotic plaques. These findings were compared with histology, and high sensitivity and specificity was calculated for calcified plaques (100 and 90%) and lipid-rich plaques (90 and 75%). 

CMR has also been correlated with findings on IVUS and it appeared that CMR was equally able to detect coronary plaques [129,130]. In addition, with CMR, the coronary wall thickness can be measured as a surrogate for the IVUS-derived plaque burden [131]. However, CMR seems to overestimate this when compared with IVUS [129]. These coronary wall measurements with CMR enabled the detection of positive vessel remodeling [132]; however, this finding has not yet been correlated with clinical outcome. Lastly, attempts have been made to measure disease activity with CMR, by evaluating the severity of contrast enhancement as a measure for inflammation and angiogenesis [133], or the presence of increased water content as a measure for edema, associated with inflammation [134].

### 4.3. Positron Emission Tomography

Positron emission tomography (PET) is a nuclear imaging modality that may be used in addition to structural imaging with CTCA or CMR. With the use of PET tracers ^18^F-fluorodeoxyglucose (^18^F-FDG) or ^18^F-sodium fluoride (^18^F-NaF), several high-risk features of the coronary plaque can be visualized.

The glucose analogue ^18^F-FDG visualizes metabolic activity, and in the setting of atherosclerosis, its uptake by plaque macrophages illustrates the presence of inflammation [135]. In carotid arteries, the extent of ^18^F-FDG uptake of the plaque was associated with increased inflammation as determined with histopathological staining [136]. With respect to vulnerable plaque detection in the coronary arteries, studies demonstrated conflicting results. Two studies reported that the ^18^F-FDG uptake was increased in culprit lesions in patients with ACS compared with non-ACS lesions [137,138]. Recently, Joshi et al. performed PET imaging with both ^18^F-FDG and ^18^F-NaF in 40 patients with recent ACS and 40 patients with stable CAD [139]. Contrarily, this study observed no difference in ^18^F-FDG uptake between culprit plaques and non-culprit plaques in the 40 patients with ACS. Coronary atherosclerosis imaging with ^18^F-FDG is limited by the uptake of the tracer by the myocardium, which obscures the uptake of the coronary arteries. Indeed, more than half of the ^18^F-FDG scans could not be analyzed because of myocardial tracer uptake. Moreover, ^18^F-FDG imaging requires specific patient preparation and diet restrictions hampering ad hoc analysis in patients with ACS.

**Figure 7:**
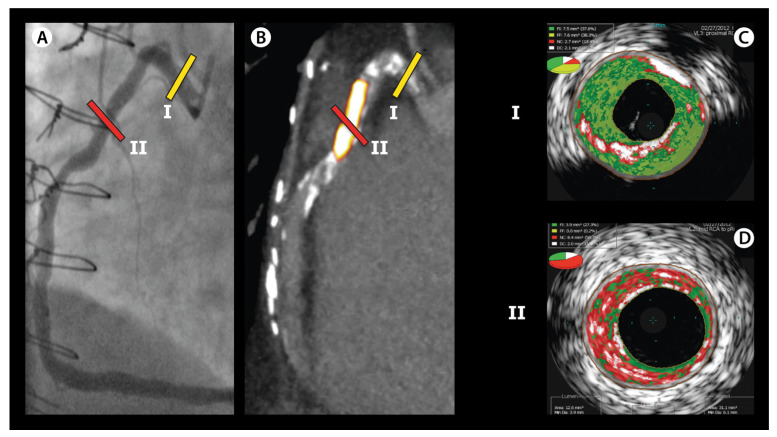
Vulnerable plaque on ^18^F-NaF PET imaging. In (**A**), coronary angiography demonstrates two non-obstructive lesions in the proximal and mid-right coronary artery in a patient with stable angina. (**B**) demonstrates the corresponding ^18^F-NaF PET-CT image, which indicates no uptake of ^18^F-NaF in lesion I, but an increased uptake in lesion II. (**C**) and (**D**) demonstrate the corresponding radiofrequency IVUS images. Lesion I (**C**) appears to be a lesion consisting of fibrous tissue (green) with confluent calcium (white) with acoustic shadowing. However, the ^18^F-NaF positive lesion II (**D**) appears to consist of a large necrotic core (red), with microcalcifications (white), suggestive for a vulnerable plaque. Image adapted from Joshi NV, et al. [139] CC-BY [4.0].

On the other hand, ^18^F-NaF was also used in the study by Joshi et al. and it was found that this tracer was able to distinguish culprit lesions from non-culprit lesions in the 40 patients with ACS [139]; Figure 7. ^18^F-NaF is not hampered by myocardial uptake, and it visualizes microcalcifications (i.e., calcifications of <1 mm [140]) which are considered to be an important predictor for the vulnerable plaque. The results of the ^18^F-NaF PET imaging were compared with IVUS, and increased tracer uptake was associated with more high-risk plaque features on IVUS: plaques more frequently demonstrated positive remodeling, microcalcifications, and a necrotic core. Hence, ^18^F-NaF seems a promising tracer for the detection of vulnerable plaques with PET, and the prognostic significance is currently tested in a large prospective study (PREFFIR study, NCT02278211). Additionally, other PET tracers that are already established in oncology could perhaps be more specific for vascular inflammation and are currently under research for imaging of the vulnerable plaque, such as ^68^Ga-DOTATATE, ^18^F-FMCH, and ^11^C-PK11195 [135].

### 4.4. Hybrid Imaging

Hybrid imaging combines information from multiple imaging modalities, resulting in a visual representation of both anatomical as well as functional characteristics such as disease activity or hemodynamics [141]. The most typical form of hybrid non-invasive imaging for coronary plaque assessment is the combination of CTCA (or less commonly CMR) with molecular imaging such as PET or single positron emission computed tomography. This offers both structural information about plaque morphology and volume, as well as the ability to differentiate between quiescent and active disease [142]. The aforementioned hybrid modality that combines CTCA with fractional flow reserve is discussed previously and is especially useful to evaluate whether certain coronary plaques cause hemodynamic significant flow-limitation, although it may also be used in the vulnerable plaque assessment.

**Figure 8:**
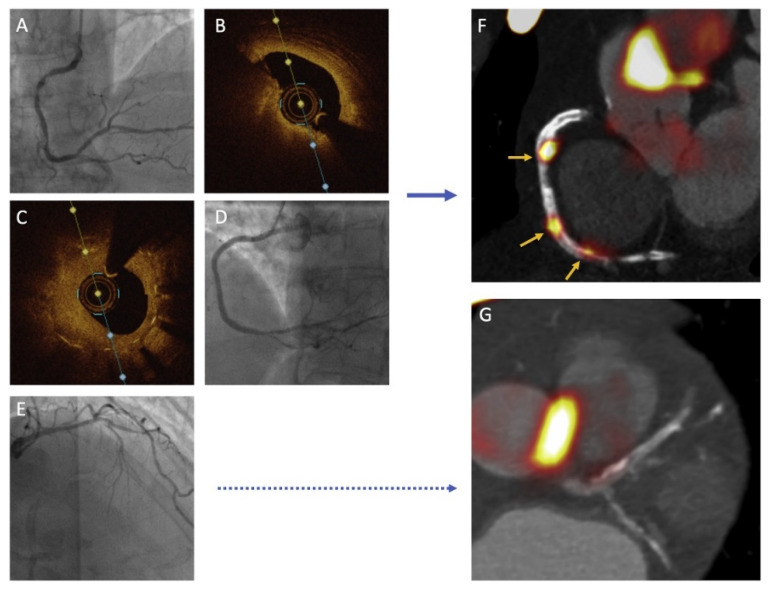
Hybrid imaging in a patient presenting with recurrent non-ST-segment elevation ACS. (**A**) demonstrates severe in-stent restenosis in the proximal right coronary artery (RCA) with de novo lesion in the mid-RCA; (**B**) OCT demonstrates plaque rupture in the mid-RCA and (**C**) severe neointimal hyperplasia in the previously placed stent in the proximal RCA. (**D**) demonstrates the result of successful revascularization, with remaining diffuse disease in the left coronary artery (**E**). (**F**,**G**) demonstrate the ^18^F-NaF PET-CT images with high ^18^F-NaF uptake in the culprit artery, especially in the culprit lesion in the proximal RCA. The left coronary artery demonstrates no uptake of the radioactive tracer. Reprinted from Bing et al. [141], with permission from Elsevier.

Additionally, combining non-invasive functional imaging with coronary angiography and intracoronary imaging could have potential benefit in providing details about the type of plaque and state of disease activity. Bing et al. [141] present a case where ^18^F-NaF PET-CT was performed in a patient with recurrent non-ST-segment elevation ACS based on in-stent restenosis of the 6-month old stent; Figure 8. OCT during the procedure demonstrated aggressive in-stent neointimal hyperplasia in the culprit lesion, as well as plaque rupture distally from the culprit. In the left coronary artery, diffuse atherosclerosis was observed. ^18^F-NaF PET-CT distinguished the high-risk plaques in the culprit artery, with the culprit lesion demonstrating the highest ^18^F-NaF uptake, from the stable, PET-negative plaques in the non-culprit artery. This could guide the treatment strategy for non-culprit plaques. Thus, the interest for these hybrid imaging modalities is clear, although its clinical application is currently limited by high costs and low availability.

## 5. Clinical Implications

As indicated in the consensus document by Tomaniak et al. [143], challenges for the future lie in improving the available imaging techniques and in establishing potential treatment options. First, a significant portion of ACS arises from plaque erosion instead of plaque rupture. Plaque erosion typically results from plaques with other high-risk features, possibly warranting different imaging definitions or modalities. Second, Tomaniak et al. [143] argue that the current positive predictive value for vulnerable plaques of the available imaging modalities is too low for application in routine clinical practice, but that improvement of these modalities is contributory for the development of future pharmacological and local treatment strategies. The support of machine learning methods or hybrid imaging modalities could potentially be useful in this regard.

To implement the imaging modalities mentioned in this review as a screening tool to guide primary or secondary prevention therapies, it should be elucidated whether vulnerable plaque features are modifiable and if plaque rupture can herewith be prevented. Large studies on lipid-lowering agents, both statins and proprotein convertase subtilsin-kexin type 9 (PCSK9) inhibitors, demonstrated that strict lipid-lowering leads to plaque stabilization by increasing the fibrous cap thickness and reducing the plaque burden and inflammation [144,145,146,147,148,149,150,151,152,153,154]. These lipid-lowering agents reduced the frequency of cardiac events [155,156]. In addition, anti-inflammatory agents, such as colchicine, studied in the LoDoCo2 trial and COLCOT trial [157,158], and the interleukin-1-beta antagonist canakinumab, studied in the CANTOS trial [159], are able to reduce the risk for cardiac events.

In addition, local percutaneous treatment with bioresorbable vascular scaffold (BVS) has recently been studied in the PROSPECT-ABSORB study [160]. Patients underwent NIRS-IVUS after successful treatment of all coronary lesions and lesions with an IVUS-derived plaque burden of ≥65% were randomized to treatment with the ABSORB BVS or optimal medical therapy. The minimal luminal area was significantly greater at 25 months follow-up in patients treated with ABSORB BVS compared with patients only on medical therapy. No difference in complications was observed, indicating that local treatment of the vulnerable plaques was safe. However, no effect was observed for target lesion failure between groups; thus, there seems to be no benefit from stenting with ABSORB BVS to prevent future events. Future studies are necessary to demonstrate clinical benefit from local treatment. The ABSORB BVS has been retracted from the market, resulting in a premature halt of the PECTUS study which investigated the potential clinical benefit of treatment of vulnerable plaques with ABSORB BVS compared with medical therapy [161]. The PREVENT trial (NCT02316886) is currently ongoing, which will compare stenting of a vulnerable plaque with drug-eluting stent or BVS with optimal medical treatment only. However, since stenting comes with stent-related problems, treatment of vulnerable plaques with drug-coated balloon could have potential benefits, which are currently being investigated in the DEBuT-LRP study (NCT04765956).

## 6. Conclusions

Several invasive and non-invasive imaging modalities have been developed that enable the detection of vulnerable coronary plaques at risk to cause future cardiac events. Currently, IVUS and OCT, with the addition of NIRS, could serve as a screening modality in patients undergoing invasive coronary angiography. To implement the non-invasive imaging modalities for screening to guide primary prevention or secondary therapies, further studies are necessary to optimize vulnerable plaque detection and to correlate the findings with invasive imaging and clinical outcome. The developments in imaging modalities facilitate studies investigating treatment possibilities for vulnerable plaques to modify the high-risk plaque features and reduce the subsequent cardiovascular risk. Systemic treatment with lipid-lowering and anti-inflammatory agents stabilizes vulnerable plaques and reduces the cardiovascular risk. Local percutaneous treatment options seem safe, but future clinical studies are necessary to confirm their clinical benefit.

## Figures and Tables

**Table 1:** Invasive imaging modalities.

	Intravascular Ultrasound (IVUS)	Optical Coherence Tomography (OCT)	Near-Infrared Spectroscopy (NIRS)	Angioscopy	Thermography
**Technical features**					
Spatial resolution	20–100 μm	10–15 μm	-	-	0.5 mm
Tissue penetration, **mm**	4–8	1–2	1–2	0	N/A
Pullback speed, **m/s**	1–10	20	0.5–1.0	N/A	N/A
**Detection of vulnerable plaque features**					
Lipid-rich core	++	+	+++	++	−
Fibrous cap thickness	−	+++	−	+	−
Calcifications	+++	+	−	−	−
Positive vessel remodeling	+++	−	−	−	−
Inflammation	−	+	−	−	+++
Neovascularization	−	+	−	−	−
Intraplaque hemorrhage	+	+	−	−	−

N/A indicates not applicable. Indicator − means not distinguishable; + barely distinguishable; ++ moderately distinguishable; +++ well delineated.

**Table 2:** Non-invasive imaging modalities.

	Computed Tomography Coronary Angiography (CTCA)	Cardiovascular Magnetic Resonance (CMR)	^18^F-FDG Positron Emission Tomography (PET)	^18^F-NaF Positron Emission Tomography (PET)
**Technical features**				
Spatial resolution, **mm**	0.4	0.5–1	4–5	4–5
Radiation exposure	Yes	No	Yes	Yes
Iodine contrast	Yes	No	No	No
**Detection of vulnerable plaque features**				
Lipid-rich core	++	+++	−	−
Fibrous cap thickness	−	−	−	−
Microcalcifications *	−	−	−	+++
Spotty calcifications **	+++	++	−	−
Positive vessel remodeling	+++	+	−	−
Inflammation	−	+	+++	−
Neovascularization	−	++	−	−

* Microcalcifications are calcifications < 1 mm; ** Spotty calcifications are calcifications < 3 mm. Indicator—means not distinguishable; + barely distinguishable; ++ moderately distinguishable; +++ well delineated.

## Data Availability

Data are available upon reasonable request.
